# High-Performance Photocatalytic Multifunctional Material Based on Bi_4_Ti_3_O_12_-Supported Ag and Ti_3_C_2_T_x_ for Organic Degradation and Antibacterial Applications

**DOI:** 10.3390/bios15010011

**Published:** 2024-12-31

**Authors:** Kexi Zhang, Bingdong Yan, Xiaohong Wang, Yang Cao, Wanjun Hao, Jinchun Tu

**Affiliations:** 1 State Key Laboratory of Marine Resource Utilization in South China Sea, School of Materials Science and Engineering, Hainan University, Haikou 570228, China; zhangkexi@hainanu.edu.cn (K.Z.); wangxiaohong@hainanu.edu.cn (X.W.); cy507@hainanu.edu.cn (Y.C.); 2 College of Science, Laboratory of Child Cognition & Behavior Development of Hainan Province, Qiongtai Normal University, Haikou 571127, China; yanbingdong@outlook.com

**Keywords:** bismuth titanate, MXene, multifunctional application, photodegradation, antibacterial properties

## Abstract

With the rapid development of modern science and technology and the diversification of social needs, traditional single-performance materials struggle to meet the complex and changeable application scenarios. To address the multifaceted requirements of biomedical applications, such as disease diagnosis and treatment, scientists are dedicated to developing new multifunctional biomaterials with multiple activities. Bi_4_Ti_3_O_12_ (BTO), despite its versatility and application potential, has insufficient photocatalytic performance. Silver nanoparticles (Ag) and Ti_3_C_2_T_x_ are particularly effective as antibacterial materials but they have relatively single functions. In this study, BTO/Ag/Ti_3_C_2_T_x_ biomultifunctional materials were constructed by combining BTO with Ag and Ti_3_C_2_T_x_. We discovered that the addition of Ag and Ti_3_C_2_T_x_ effectively optimized the visible light absorption characteristics of BTO, reduced the electron transfer resistance, and increased the carrier concentration, thus significantly improving the photocatalytic performance of composite material, thereby markedly improving the composite’s photocatalytic performance and its efficacy in photochemical sensing and photodegradation. At the same time, BTO, as a carrier, effectively avoids Ag and Ti_3_C_2_T_x_ agglomeration and gives full play to its antibacterial properties. In the specific performance studies, ascorbic acid and MB were used as the subjects of photochemical sensing and photodegradation properties, while *Escherichia coli* and *Staphylococcus aureus* were tested for antibacterial properties. The BTO/Ag/Ti_3_C_2_T_x_ composite showed remarkable results in all assessments, demonstrating its potential as a versatile antibacterial and photocatalytic material.

## 1. Introduction

The rapid evolution of modern technology and the increasing diversity of societal needs have outpaced the capabilities of traditional single-performance materials to meet the demands of complex and variable application scenarios [1]. As life science, materials science, and medical engineering become more deeply intertwined, scientists have begun to develop new materials that are biocompatible, biodegradable, and diverse and bioactive to meet the needs of complex biomedical applications, such as disease diagnosis and treatment [2]. In this context, the emergence of multifunctional biomaterials has become a trending topic of research within materials science. Bismuth titanate (Bi_4_Ti_3_O_12_) is an extremely competitive candidate for photocatalysis due to its unique crystal and electron structures. As a member of Aurivillius family compounds, Bi_4_Ti_3_O_12_ has a layered perovskite structure built up by [Bi_2_O_2_]^2+^ fluorite-type and [Bi_2_Ti_3_O_10_]^2−^ perovskite-type layers. The [Bi_2_Ti_3_O_10_]^2−^ layer is composed of three TiO_6_ octahedral layers with Bi^3+^ at an a-site, which are sandwiched between the [Bi_2_O_2_]^2+^ layers along the c-axis [3]. This arrangement can create an internal electric field that facilitates the separation of the electron hole pairs.

However, the inherent band structure of bismuth titanate materials results in lower visible light absorption, a higher carrier recombination rate, and lower carrier energy state, which limits their application in photocatalysis [4]. Despite these limitations, by loading noble metal nanoparticles on semiconductor materials, its visible light absorption can be optimized. Ag has been extensively studied as a common antimicrobial material. It is reported that nano silver mainly disrupts the synthesis of the cell wall, damages the cell membrane, suppresses the cell protein synthesis, interferes with the synthesis of nucleic acid, and combines with the sulfur-containing hydrogen group (-SH) in bacteria, causing microorganisms to lose activity and eventually die [5]. In addition, Ag can also be used as a photosensitized material to effectively improve the comprehensive performance of photocatalytic materials, showing unique application potential in photocatalysis and other fields. The surface loading of metallic Ag nanoparticles on some semiconductors has been shown to enhance their photocatalytic performance in the visible region. Moreover, the insertion of Ag reduces the recombination rate of charge carriers and improves photocatalytic activity. However, due to limitations in synthesis techniques, only a limited amount of silver nanoparticles can be introduced onto the surface of semiconductor materials [6,7], resulting in margina enhancement in surface catalytic performance, visible light absorption performance, and charge separation performance of the semiconductor material’s active sites. To address this, researchers are exploring the introduction of materials with more abundant surface active sites, stronger visible light absorption ability, and better compatibility with semiconductor materials [8].

Transition metal carbides or nitrides (MXenes) are regarded as next-generation two-dimensional (2D) materials for various applications, such as photocatalysis, electrocatalysis, supercapacitor, lithium-ion battery, and biomedicine, owing to their unique physicochemical properties [9]. In photocatalysis, MXenes facilitate fast photogenerated charge carrier separation and provide abundant surface functional groups for light harvesting materials, rendering high photoconversion efficiency achievable. The antibacterial activity of MXenes has been associated with the production of reactive oxygen species (ROS) and direct contact with the bacteria membrane, penetrating into the bacteria and interacting with sulfur-containing proteins, as well as phosphorus-containing DNA, leading to bacterial cell death. Therefore, enormous theoretical and experimental studies have recently demonstrated the potential of MXene invarious photocatalytic applications [10].

**Scheme 1:**
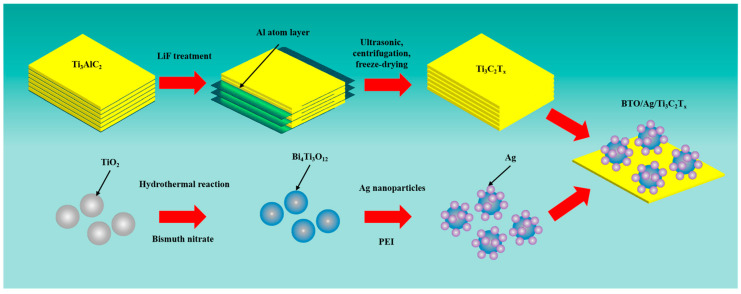
Schematic of the material synthesis.

To develop multifunctional biological materials with excellent performance, this study integrated BTO with Ag and Ti_3_C_2_T_x_ to construct a BTO/Ag/Ti_3_C_2_T_x_ composite. As shown in Scheme 1, BTO nanoparticles were successfully prepared via TiO_2_ hydrothermal conversion. The Ag nanoparticles were decorated onto the surface of the BTO, and after binding with Ti_3_C_2_T_x_, a Schottky heterojunction was successfully constructed. The significant intrinsic difference in Fermi energy levels between the BTO to Ag and Ti_3_C_2_T_x_ is believed to be the primary source of the photoelectrons driving force in this photochemical system. Investigations into photodegradation revealed that the incorporation of Ag and Ti_3_C_2_T_x_ effectively optimized the visible light absorption, reduced the electron transfer resistance, and improved the carrier concentration of BTO/Ag/Ti_3_C_2_T_x_. These enhancements subsequently bolstered the photocatalytic performance of BTO/Ag/Ti_3_C_2_T_x_ and improved the photodegradation performance. In terms of antibacterial properties, it was found that BTO served as an effective carrier for Ag and Ti_3_C_2_T_x_, preventing their agglomeration and thus maximizing the antibacterial properties. The design strategy of composite materials in this paper provides innovative insights for the preparation of novel multifunctional photocatalytic materials.

## 2. Materials and Methods

### 2.1. Materials, Reagents, and Apparatus

All reagents were used directly without purification. Titanium dioxide (TiO_2_), bismuth nitrate (Bi(NO_3_)_3_), dihydrate triodium citrate (C_6_H_5_Na_3_O_7_), silver nitrate (AgNO_3_), sodium borohydride (NaBH_4_), polyethylenimine (PEI), hydrofluoric acid (HCl), hydrochloric acid (HF), and lithium fluoride (LiF) were purchased from Sinopharm Chemical Reagent Co., Ltd. (Shanghai, China).

The XRD patterns of the as-prepared films were recorded on a Philip X’pert X-ray diffractometer (PANalytical, Almelo, The Netherlands; CuKa irradiation, λ = 0.15418 nm) from 5° to 80° at a scanning speed of 10°/min. The morphology of samples was characterized via field-emission scanning electron microscopy (FESEM, Hitachi SU8010, Tokyo, Japan). Transmission electron microscopy (TEM) images were obtained via TEM (JEOL JEM 2100, Tokyo, Japan). UV–vis absorption spectra were obtained on a UV–vis absorption spectrophotometer (UV–vis, Thermo Fisher Evolution 220, Thermo Fisher Scientific, Waltham, MA, USA). PL measurements were made on an FL3-P-TCSPC time-resolved fluorescence spectrometer (Horiba Jobin Yvon, Longjumeau, France). PEC tests were performed with a homemade PEC system. A xenon lamp (wavelength range: 200–1000 nm, Beijing Ceaulight Technology Co., Ltd., Beijing, China) served as the irradiation source. The photocurrent was recorded on an electrochemical workstation (Chenhua Technology Co., Ltd., Shanghai, China) by a three-electrode electrochemical system with Ag/AgCl reference electrodes and 0.5 cm × 0.5 cm Pt foil as counter electrodes under off-on-off (20 s-20 s-20 s) switching light at 0.0 V in the electrolyte of 0.1 M PBS (containing 0.1 M AA, pH 7.4) [11]. Electrochemical impedance spectroscopy (EIS) was performed on a Bio-Logic electrochemical workstation (France) in a 0.1 M KCl solution containing 5.0 mM K_3_[Fe(CN)_6_]/K_4_[Fe(CN)_6_] (1:1) at an open circuit potential over a frequency range of 10 mHz–100 kHz with 5 mV of AC perturbation.

### 2.2. Synthesis of the BTO

Here, 0.4 g of TiO_2_ and bismuth nitrate solution (20 mL, 3 mol·L^−1^) was added and sonicated for 15 min. The resulting suspension was aged at room temperature for 12 h, filtered, and dried at 80 °C for 12 h. The resultant complex was heated to 600 °C in a 2 °C·min^−1^ tubular furnace and stored at this temperature for 2 h and naturally cooled down to room temperature.

### 2.3. Synthesis of Ag Nanoparticles

Here, 20 mL of a 1 wt% mass concentration of dihydrate triodium citrate was added to 75 mL of distilled water and heated to 70 °C, followed by additional stirring for 15 min. Subsequently, a 1 wt% mass concentration of silver nitrate solution and 2 mL of a 0.1 wt% new sodium borohydride solution were added, and the mixture was vigorously stirred at 70 °C for 1 h and cooled to room temperature, and the volume was adjusted to 100 mL.

### 2.4. Fabrication of BTO/Ag

Here, 0.25 g of PEI was dissolved in 50 mL of distilled water and stirred for 10 min; then, 0.2 g of bismuth titanate was added for 2 h, washed via centrifugation, and redispersed in 10 mL of distilled water. Take 1 mL of PEI-bismuth titanate in a 10 mL dispersion, add 10 mL of silver particle solution (4 nm), sonicate for 1 h, wash via centrifugation, and disperse in 10 mL of PBS buffer. Parametric tuning experiments for the Ag nanoparticle content are provided in the Supporting Information.

### 2.5. Preparation of Ti_3_C_2_T_x_ MXene

The concentration of 49% HF, 20 mL of concentrated hydrochloric acid, and 10 mL of deionized water were added to the polytetrafluoroethylene lining and placed in a water bath. When the temperature reached 35 °C, a small amount of 2 g of Ti_3_AlC_2_ was added slowly. After 12 h, the liquid was poured into a centrifuge tube for centrifugation and cleaning, and then, this was repeated 5 to 6 times until the supernatant pH reached neutrality and the precipitate was collected. Then, 2 g of LiF was dissolved in 40 mL of deionized water as an intercalation agent. The precipitate was poured into the intercalator, fully shaken, and stirred for 12 h at room temperature. After intercalation, it was centrifuged with deionized water and then sonicated in an ice water bath for 30 min. The supernatant was collected via centrifugation and finally freeze-dried to obtain the Ti_3_C_2_T_x_ MXene powder.

### 2.6. Fabrication of BTO/Ag/Ti_3_C_2_T_x_

Here, 50 mg of pre-prepared Ti_3_C_2_T_x_ MXene powder was dissolved in deionized water and sonicated for 15 min to achieve an even dispersion. Then, 50 mg of BTO/Ag was added to the mixture and sonicated for another 20 min for well dispersion before 6 h of stirring. The result was washed via centrifugation to obtain the BTO/Ag/Ti_3_C_2_T_x_ complex. Parametric tuning experiments for the Ti_3_C_2_T_x_ content are provided in the Supporting Information.

### 2.7. Bacterial Culture and Antibacterial Experiments

Single colonies of *E. coli* and *S. aureus* on solid agar plates were transferred to 5 mL of liquid LB culture medium and grown at 37 °C for 12 h at 200 rpm. The antibacterial efficiencies of samples were evaluated using the spread plate method. The bacteria were then diluted to 10^6^ CFU mL^−1^ with PBS. Different samples (100 μL, 200 μg·mL^−1^) were respectively mixed with bacteria (10^6^ CFU mL^−1^) in a 96-well plate. Afterward, the mixed solutions were diluted 1000 times, and its solutions (30 µL) were taken for plating on solid medium using spread plate method and cultured for 12 h before observing the number of the bacteria colonies. Image J (version 1.52a, NIH, Bethesda, MD, USA) software was used to quantify the number of bacteria.

## 3. Results

### 3.1. Composition and Morphology Characterization of the Materials

**Figure 1:**
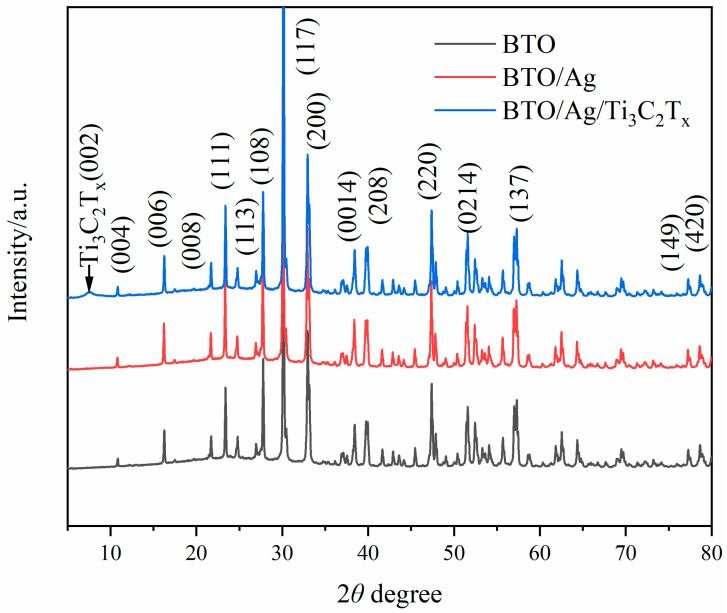
X-ray diffraction patterns of the BTO, BTO/Ag, and BTO/Ag/Ti_3_C_2_T_x_.

The composition of samples was investigated via X-ray diffraction (XRD). As shown in Figure 1, BTO showed peaks at 16.2°, 21.6°, 23.3°, 24.6°, 27.3°, 30.1°, and 32.9°, which were consistent with Bi_4_Ti_3_O_12_ (006), (008), (111), (113), (108), (117), and (200) (JCPDS No. 72-1019). The BTO/Ag sample maintained the same diffraction patterns as Bi_4_Ti_3_O_12_. However, the characteristic peak of Ag was not observed in the BTO/Ag sample, likely due to the small amount of Ag [12]. The BTO/Ag/Ti_3_C_2_T_x_ composite exhibited new diffraction peaks at 7.4°, which were assigned to the Ti_3_C_2_T_x_ (002) diffraction peaks. The observation is associated with the increase in the vertical axis spacing within the Ti-C layer after the etching of Al atoms in theTi_3_AlC_2_ structure [13].

**Figure 2:**
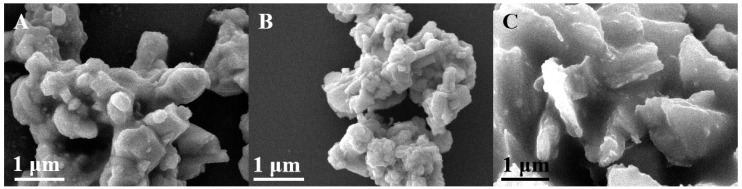
FESEM images of (**A**) BTO, (**B**) BTO/Ag, and (**C**) BTO/Ag/Ti_3_C_2_T_x_.

The surface morphology of the sequentially synthesized materials is displayed in Figure 2. Figure 2A,B show the morphology of the BTO and BTO/Ag, while Figure 2C shows the morphology of the BTO/Ag/Ti_3_C_2_T_x_. The BTO appears as irregular particles composed of many smaller particles. The BTO/Ag exhibits a similar morphology than BTO but with a noticeably rougher surface, suggesting the presence of Ag nanoparticles on the surface of BTO. Upon the addition of Ti_3_C_2_T_x_ to the system, the material’s morphology takes on a distinct multi-layered block structure, indicating the successful loading of Ti_3_C_2_T_x_ onto the BTO/Ag.

**Figure 3:**
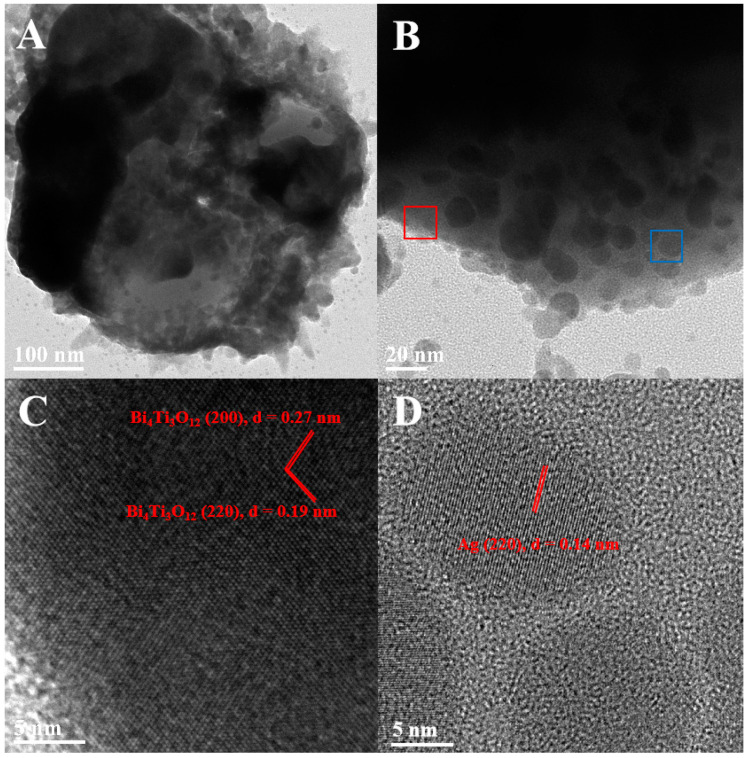
(**A**,**B**) TEM image of BTO/Ag/Ti_3_C_2_T_x_ and (**C**,**D**) HRTEM image of the BTO and Ag (image (**C**) is a zoom of the red box in image (**B**), and image (**D**) is a zoom of the blue box in image (**B**).

The crystallographic information of the BTO/Ag/Ti_3_C_2_T_x_ is shown in Figure 3. Figure 3A shows an irregular structure, and the enlarged view in Figure 3B reveals a large number of spherical nanoparticles on the surface of BTO, suggesting that the silver nanoparticles have been loaded well onto the surface of BTO. The high-resolution transmission electron microscope (HRTEM) images of BTO in Figure 3C show clear crystalline lattice fringes with lattice spacings of 0.27 nm and 0.19 nm matching well with the (002) and (202) planes of orthorhombic Bi_4_Ti_3_O_12_, respectively [14]. Figure 3D depicts Ag particles of about 10 nm with a clear lattice space of 0.223 nm, aligning with (002) and (202) lattice planes of orthorhombic Bi_4_Ti_3_O_12_ [15]. The use of PEI facilitates better anchoring of Ag on the surface of BTO and helps to maintain the nanoparticle’s good monodispersity.

**Figure 4:**
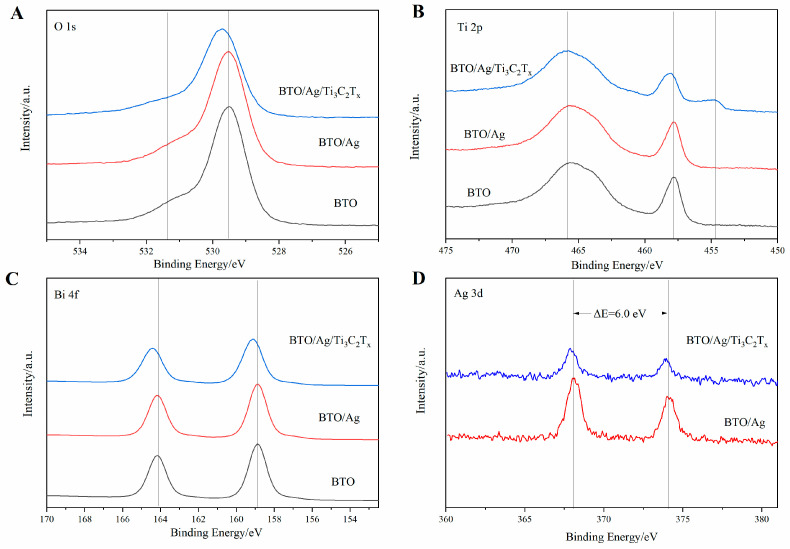
XPS exact spectrum of BTO, BTO/Ag, and BTO/Ag/Ti_3_C_2_T_x_: (**A**) is the spectrum of the 1s orbit of O, (**B**) is the spectrum of the 2p orbit of Ti, (**C**) is the spectrum of the 4f orbit of Bi, and (**D**) is the spectrum of the 3d orbit of Ag.

To explore the chemical environment of the elements in BTO and the interaction with Ag and Ti_3_C_2_T_x_, X-ray photoelectron spectroscopy (XPS) was used for the characterization of BTO, BTO/Ag, and BTO/Ag/Ti_3_C_2_T_x_, with the results shown in Figure 4. Initially, the XPS spectrum of O 1s (Figure 4A) was analyzed. The peaks in the O 1s spectrum near 531 eV and 529 eV correspond to defective oxygen and lattice oxygen, respectively. The O 1s peak of BTO/Ag/Ti_3_C_2_T_x_ is red-shifted after recombination with Ti_3_C_2_T_x_, indicating that Ti_3_C_2_T_x_ increases the electron density of O atoms on BTO. This implies that Ti_3_C_2_T_x_ donates electrons to BTO/Ag, potentially promoting the conversion of Ag to Ag^+^, enhancing the antibacterial properties. Further studies show that defective oxygen constituted 21.7% of oxygen elements in BTO, decreasing to 11.0% in BTO/Ag/Ti_3_C_2_T_x_. This suggests that the use of Ti_3_C_2_T_x_ can appropriately reduce oxygen defects in BTO, thereby reducing electron scattering and benefits for the faster electron transfer kinetics.

Comparing the XPS results of BTO and BTO/Ag, there was a significant change in the peak shape of Ti 2p (Figure 4B) and Bi 4f (Figure 4C) in the BTO/Ag/Ti_3_C_2_T_x_ spectrum, which indicate that the modification of Ti_3_C_2_T_x_ had a noticeable effect on the crystal structure of BTO. A further analysis reveals a blue shift of the Ti 2p and Bi 4f spectrum after Ti_3_C_2_T_x_ modification, probably due to the increase in the electron density of oxygen atoms (Figure 4B,C). This increased state density can influence bismuth atoms through chemical bonding, thus increasing the electron density of Bi [16,17]. Considering that bismuth titanate is stacked alternately by the perovskite layer and the bismuth oxygen layer and the blue shift of Bi 4f is more pronounced than Ti 2p, it can be inferred that the Ti_3_C_2_T_x_ mainly interacts with the bismuth oxygen layer. To determine the chemical status of Ag in BTO/Ag and BTO/Ag/Ti_3_C_2_T_x_, the XPS spectrum of Ag 3d (Figure 4D) was analyzed. According to the test results, only two sharp symmetrical peaks appear in the Ag 3d spectrum, and the energy difference between the two peaks is 6.0 eV, indicating that the chemical state of Ag is single and simple, which suggests that a silver element exists in a single form in both BTO/Ag and BTO/Ag/Ti_3_C_2_T_x_ [18].

### 3.2. Light Absorption and Band Gap Width Characterization of the Materials

**Figure 5:**
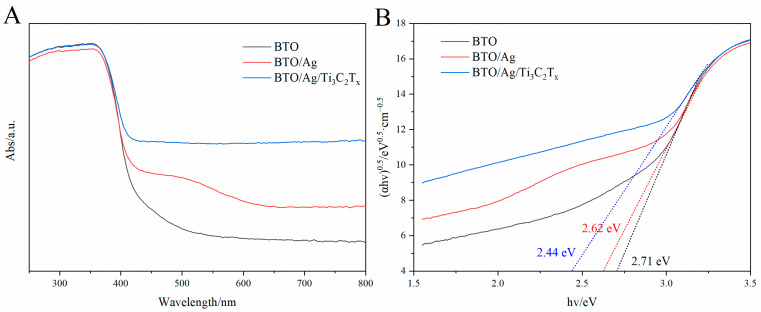
(**A**) Optical absorption spectra and (**B**) plot showing the square root of the measured absorption coefficient as a function of the photon energy of BTO, BTO/Ag, and BTO/Ag/Ti_3_C_2_T_x_.

The UV diffuse reflection spectrum was used to study the gap width and light absorbance of BTO, BTO/Ag, and BTO/Ag/Ti_3_C_2_T_x_. As depicted in Figure 5A, the introduction of Ag and Ti_3_C_2_T_x_ greatly improves the light-absorption capacity of the material in the visible light range without compromising its absorption in the UV region. Figure 5B shows that the gap widths of BTO, BTO/Ag, and BTO/Ag/Ti_3_C_2_T_x_ are 2.71 eV, 2.62 eV, and 2.44 eV, respectively. This result implies that BTO/Ag/Ti_3_C_2_T_x_ and BTO/Ag have markedly smaller gap widths than BTO. In addition, Figure 5A reveals that in the UV region, BTO/Ag and BTO/Ag/Ti_3_C_2_T_x_ have a better light-absorption capacity compared to BTO. The smaller band gap width and enhanced UV light absorption of BTO/Ag/Ti_3_C_2_T_x_ contribute to its higher photoelectric absorption efficiency, thereby improving its photocatalytic capacity [19]. In addition, the UV diffuse reflection spectrum test of composites with different amounts of Ag on BTO/Ag and of the amount of BTO/Ag on Ti_3_C_2_T_x_ is supplemented in the Supporting Information. The light-absorption capacity of BTO/Ag and BTO/Ag/Ti_3_C_2_T_x_ increases with the increase in the content of Ag nanoparticles and BTO/Ag added into the reaction system, but when it reaches a certain amount, the light absorption of composites begins to decline. This phenomenon is probably because of the limited loading site of BTO and Ti_3_C_2_T_x_ for Ag nanoparticles and BTO/Ag, respectively.

### 3.3. PEC Characterization of the Materials

**Figure 6:**
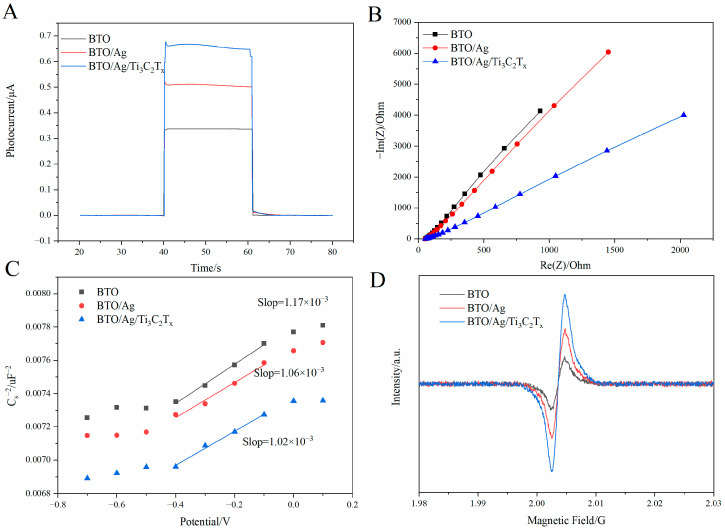
(**A**) Photocurrent, (**B**) electrochemical impedance spectroscopy, (**C**) Mott-Schottky plot, and (**D**) EPR spectrum of the BTO, BTO/Ag, and BTO/Ag/Ti_3_C_2_T_x_.

The photoelectric current responses of the materials are depicted in Figure 6A. The photocurrent of the BTO is ~0.35 μA. The growth of Ag onto the BTO led to a further increase in the photocurrent to about 0.50 μA, attributed to the surface plasmon resonance effect of Ag NPs. After the decomposition of Ti_3_C_2_T_x_, the photocurrent noticeably rose to ~26 μA, which indicated that the introduction of Ti_3_C_2_T_x_ greatly increases the photoelectric conversion efficiency of the materials.

**Figure 7:**
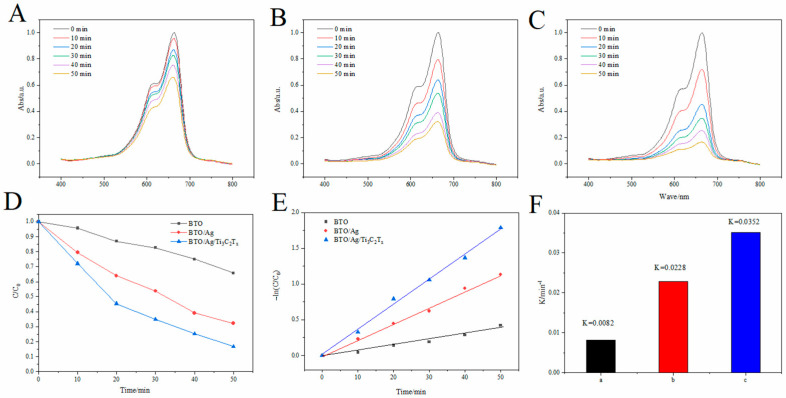
Photocatalytic degradation of MB aqueous solution over the materials: (**A**–**C**) UV-vis absorption spectra of the MB solution photodegraded by BTO, BTO/Ag, and BTO/Ag/Ti_3_C_2_T_x_, respectively; (**D**) time-dependent degradation curve of MB and (**E**) its kinetic curve over BTO, BTO/Ag, and BTO/Ag/Ti_3_C_2_T_x_; (**F**) first-order reaction rate constants of the MB degradation over the BTO, BTO/Ag, and BTO/Ag/Ti_3_C_2_T_x_ samples.

Electrochemical impedance spectroscopy (EIS) was used to compare the electron transfer resistance of BTO, BTO/Ag, and BTO/Ag/Ti_3_C_2_T_x_. As observed in Figure 6B, the impedance arc radius of BTO/Ag/Ti_3_C_2_T_x_ is less than that of BTO and BTO/Ag [20]. These results indicate that the modification of Ag and Ti_3_C_2_T_x_ can effectively reduce the electron transfer resistance of the material, which may be due to the high conductivity of the Ag nanoparticles and Ti_3_C_2_T_x_. To explore the difference in carrier concentrations between BTO and BTO/Ag, Mott-Schottky (M-S) plots of the BTO, BTO/Ag, and BTO/Ag/Ti_3_C_2_T_x_ were analyzed, as shown in Figure 7C. BTO/Ag/Ti_3_C_2_T_x_ has a lower slope in the linear region of the M-S plot, indicating a higher carrier density [1]. Given the inverse ratio between the carrier density and slope of the material, it is estimated that the carrier densities of BTO/Ag and BTO/Ag/Ti_3_C_2_T_x_ are 1.04 and 1.15 times that of BTO, respectively. The modification of Ag nanoparticles and Ti_3_C_2_T_x_ increased the carrier concentration by about 4% and 15%, which is extremely beneficial to improve the photocatalytic performance of the material. The electron paramagnetic resonance (EPR) results presented in Figure 7D were used to compare the oxygen vacancy concentrations among BTO, BTO/Ag, and BTO/Ag/Ti_3_C_2_T_x_. The test results revealed that BTO/Ag/Ti_3_C_2_T_x_ has a higher oxygen vacancy concentration than BTO/Ag and BTO. According to previous studies, appropriately increasing the oxygen vacancy of the material is beneficial to improve the carrier concentration of the material, thus enhancing the photoelectric properties of the material. In conjugation with the PEC characterization results, the introduction of Ag and Ti_3_C_2_T_x_ significantly boosts the electron transfer efficiency, carrier concentration, and oxygen hole concentration of the composite material, leading to an increased photoelectric conversion efficiency.

To confirm the composite’s superior photocatalytic capability, the photodegradation performance of BTO, BTO/Ag, and BTO/Ag/Ti_3_C_2_T_x_ compared to methylene blue (MB) was evaluated, as shown in Figure 7. The absorbance changes over time using BTO, BTO/Ag, and BTO/Ag/Ti_3_C_2_T_x_ as photocatalysts are displayed in Figure 7A, B and C, respectively. The experiments were performed under the full-spectrum irradiation conditions, with samples taken every ten minutes. The maximum absorption peak of MB was located at 663 nm, which gradually decreased over time for all samples, indicating photodegradation activity. However, a careful comparison of the results in Figure 7C shows that the absorbance changed more significantly for BTO/Ag/Ti_3_C_2_T_x_, suggesting better photodegradation performance. The absorbance at 663 nm over time was used to study the reaction order of MB photodegradation. The scatter plot in Figure 7D shows that the absorbance and time, although inversely related, are nonlinear. Therefore, the reaction is not zero-order, and it is correlated with the sublassine concentration. A subsequent scatter plot of -ln(C/C_0_) versus time [21], shown in Figure 7E, demonstrates a clear linear relationship, proving that the reaction is first-order. The reaction rate constants, derived from the slope in Figure 7F, are 0.0082 s^−1^, 0.0228 s^−1^, and 0.0352 s^−1^ for BTO, BTO/Ag, and BTO/Ag/Ti_3_C_2_T_x_, respectively. Under the experimental conditions, BTO/Ag/Ti_3_C_2_T_x_ and BTO/Ag were used as the photocatalyst, and the reaction rate was 2.78 and 4.29 times higher than that of BTO. This indicates that the incorporation of Ag and Ti_3_C_2_T_x_ can effectively improve the photocatalytic performance of the material.

### 3.4. Antibacterial Performance of Materials

**Figure 8:**
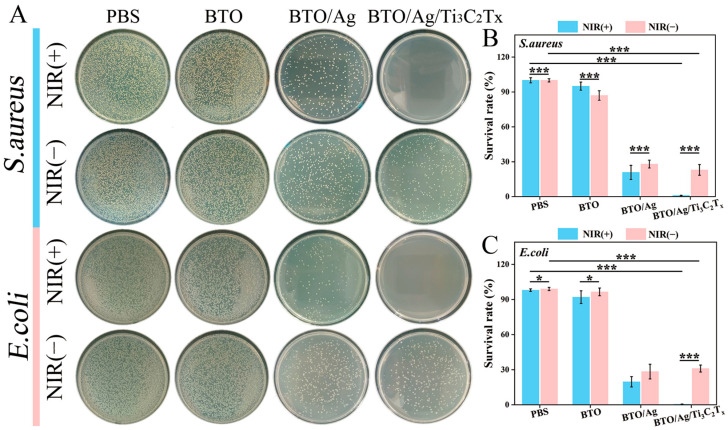
(**A**) Photograph and (**B**,**C**) colony numbers and quantitative analysis of antibacterial efficiency of *E. coli* and *S. aureus* in the presence of BTO, BTO/Ag, and BTO/Ag/Ti_3_C_2_T_x_ with (NIR+) and without (NIR−) near infrared illumination (n = 3, * *p* < 0.1, *** *p* < 0.001).

The antibacterial properties of BTO, BTO/Ag, and BTO/Ag/Ti_3_C_2_T_x_ are presented in Figure 8. *Escherichia coli* (*E. coli*) and *Staphylococcus aureus* (*S. aureus*) were selected as representative groups of Gram-negative and Gram-positive bacteria, respectively. Figure 8 shows the optical photo map of the experimental results (Figure 8A) and the data statistics (Figure 8B,C). As shown in Figure 8A,B, *S. aureus* and *E. coli* grow well on Luria-Bertani (LB) agar plates in PBS and even under single near-infrared ray (NIR, 808 nm) irradiation. When incubating bacteria with BTO or BTO/Ag, the bacteria colonies exhibit varying degrees of decline. This indicates that the addition of Ag nanoparticles greatly enhances the antibacterial ability of BTO. Notably, the bacteria survival rate in the BTO/Ag/Ti_3_C_2_T_x_ groups was nearly zero. This indicates that the addition of Ti_3_C_2_T_x_ can better amplify the synergistic effect of BTO and Ag.

**Scheme 2:**
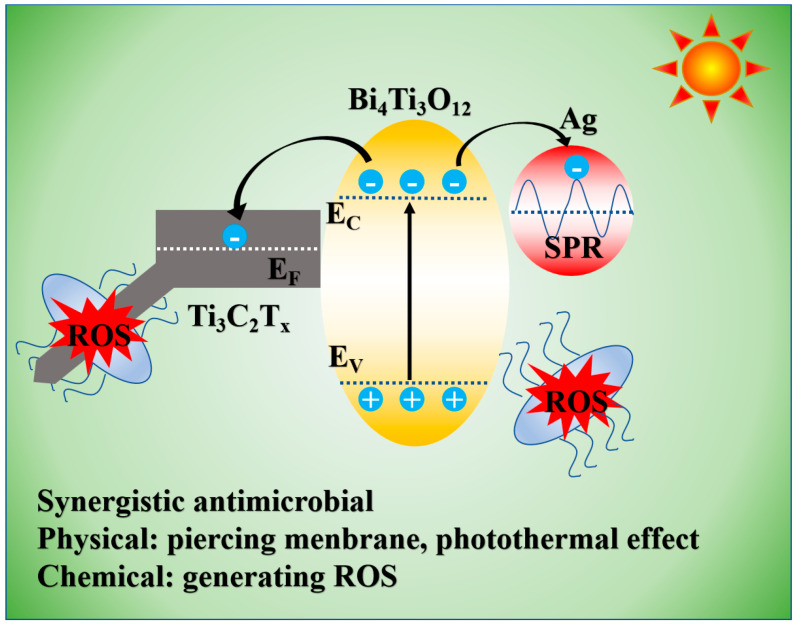
Schematic of the antibacterial materials.

As illustrated in Scheme 2, we have successfully functionalized the surface of BTO with ultra-small Ag nanoparticles and MXene nanosheets to achieve a synergistic antimicrobial effect. The photothermal effect of the MXene material itself can kill bacteria in contact. In addition, the sharp edges of the MXene nanosheets can puncture the bacterial membrane, facilitating the internalization of the conjugated Ag nanoparticles and BTO within bacteria. Meanwhile, Ag nanoparticles can release Ag^+^ ions to kill bacteria, and their own SPR effect can promote the light-absorption properties of BTO and MXene materials. This internalization enhances reactive oxygen species (ROS) generation and disturbs normal bacterial metabolism [22]. Moreover, the generated ROS are in a localized, high-concentration state, acting as an ROS reservoir that provides sustained oxidization ability to oxidize bacterial membrane lipids for enhanced membrane damage, and fragments bacterial DNA, leading to eventual bacterial death [23]. This BTO/Ag/Ti_3_C_2_T_x_ conjugation realized both physical (via MXene) and chemical (via BTO and Ag) mechanisms for enhanced synergistic antimicrobial performance on both Gram-positive and Gram-negative bacteria.

## 4. Conclusions

In this work, Ag and Ti_3_C_2_T_x_ were decorated on BTO through a straightforward method to obtain BTO/Ag and BTO/Ag/Ti_3_C_2_T_x_. The BTO/Ag/Ti_3_C_2_T_x_ composite showed excellent performance in both photocatalytic degradation and antimicrobial experiments. BTO serves as a good carrier for Ag and Ti_3_C_2_T_x_, ensuring their uniform dispersion and preventing agglomeration during use. Ag and Ti_3_C_2_T_x_ has superior antibacterial properties due to its photothermal and photodynamic effects, which is the basis of ensuring the excellent antibacterial activity of the material. As a highly efficient bactericidal material, the SPR effect of Ag nanoparticles can increase the visible light-absorption and carrier-separation efficiency of the composite material. The sharp edges of the MXene nanosheets can puncture the bacterial membrane, facilitating the internalization of the conjugated Ag nanoparticles and BTO within bacteria. In conclusion, BTO/Ag/Ti_3_C_2_T_x_, as a multifunctional biomaterial, shows excellent performance and application potential in photodegradation and antibacterial fields.

## Data Availability

Data are contained within the article.

## Supplementary Materials

The following supporting information can be downloaded at: https://www.mdpi.com/article/10.3390/bios15010011/s1, Figure S1. Photocurrent test of composites with different Ag and Ti_3_C_2_Tx contents. Figure S2. UV diffuse reflection spectrum test of composites with different amounts of Ag on BTO/Ag and of the amount of BTO/Ag on Ti_3_C_2_Tx.
