# Supplementary material for: High-Performance Photocatalytic Multifunctional Material Based on Bi4Ti3O12-Supported Ag and Ti3C2Tx for Organic Degradation and Antibacterial Applications
Source: Biosensors (Basel). 2024 Dec 31;15(1):11. doi: 10.3390/bios15010011 (PMC11763257; doi:10.3390/bios15010011)
Supplement: Supplementary file 1 [file biosensors-15-00011-s001.zip › biosensors-3261704-supplementary.pdf]

Supplementary Material

High-performance photocatalytic multifunctional material based on $Bi_4Ti_3O_{12}$ supported Ag and $Ti_3C_2T_x$ for organic degradation and antibacterial applications

1. Parametric tuning experiments for Ag and $Ti_3C_2T_x$ content

The photocurrent of the composite is the strongest when 10 mL of Ag nanoparticles and 50 mg $Ti_3C_2T_x$ are added, so we choose these content as the optimal compositional combination of the BTO/Ag and BTO/Ag/$Ti_3C_2T_x$.

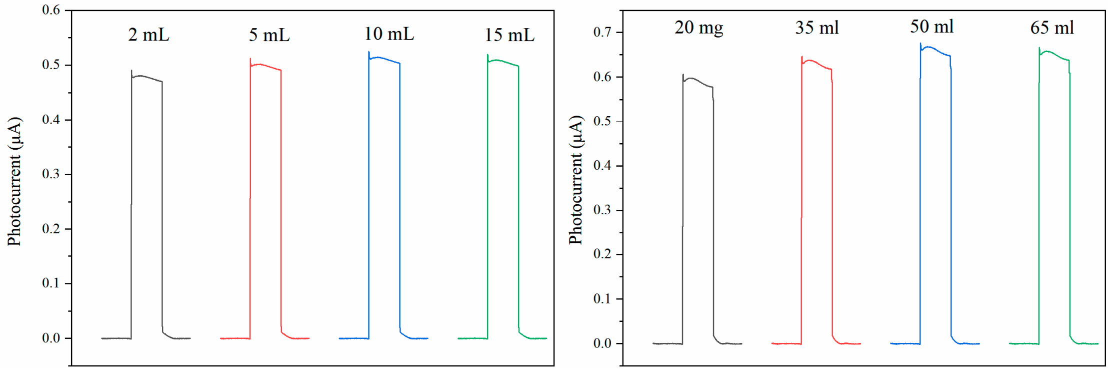

Figure S1. Photocurrent test of composites with different Ag and $Ti_3C_2T_x$ contents

2. UV diffuse reflection spectrum of composites with different amounts of Ag on BTO/Ag and of the amount of BTO/Ag

The UV diffuse reflection spectrum test of composites with different amounts of Ag on BTO/Ag and of the amount of BTO/Ag on $Ti_3C_2T_x$ is supplemented in Figure S2. The light absorption capacity of BTO/Ag and BTO/Ag/$Ti_3C_2T_x$ increases with the increase of the content of Ag nanoparticles and BTO/Ag added into the reaction system, but when it reaches a certain amount, the light absorption of composites begins to decline. This phenomenon is probably because of that the limited loading site of BTO and $Ti_3C_2T_x$ for Ag nanoparticles and BTO/Ag .

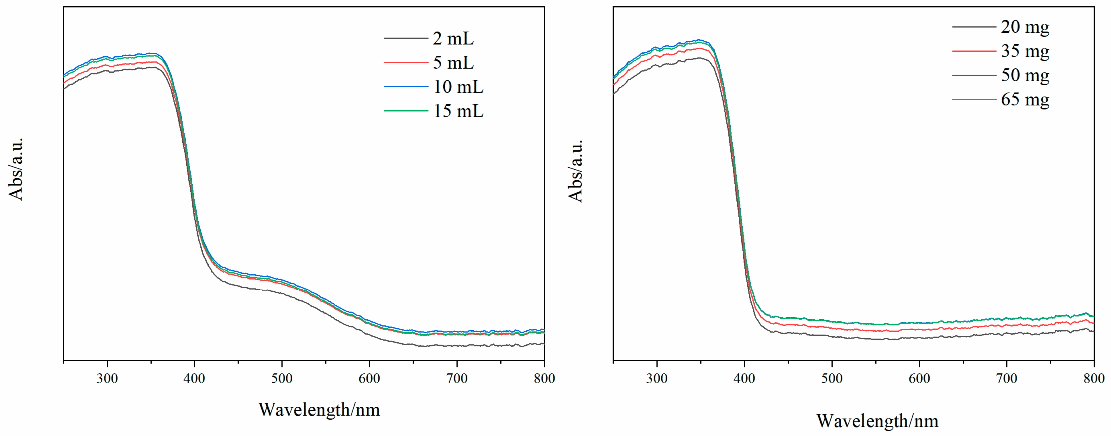

Figure S2. UV diffuse reflection spectrum test of composites with different amounts of Ag on BTO/Ag and of the amount of BTO/Ag on $Ti_3C_2T_x$
